# Bumetanide/Synthetic Saponite Composites: Performant Systems for Enhanced Drug Dissolution Rate

**DOI:** 10.3390/molecules31173015

**Published:** 2026-08-28

**Authors:** Valeria Friuli, Doretta Capsoni, Lauretta Maggi, Giovanna Bruni, Elena Cassera, Claudia Urru

**Affiliations:** 1 Department of Drug Sciences, University of Pavia, Via Taramelli 12, 27100 Pavia, Italy; valeria.friuli@unipv.it (V.F.); lauretta.maggi@unipv.it (L.M.); 2 Department of Chemistry, Physical Chemistry Section & C.S.G.I. (Consorzio Interuniversitario per lo Sviluppo dei Sistemi a Grande Interfase), University of Pavia, Via Taramelli 12, 27100 Pavia, Italy; doretta.capsoni@unipv.it (D.C.); giovanna.bruni@unipv.it (G.B.); 3 Department of Chemistry, University of Pavia, Via Taramelli 12, 27100 Pavia, Italy; elena.cassera01@universitadipavia.it

**Keywords:** synthetic saponite, bumetanide, drug–clay hybrids, dissolution test, poor water solubility, solid-state characterization

## Abstract

Bumetanide is an efficient diuretic drug with poor water solubility and dissolution rate. In this study, bumetanide/synthetic saponite systems with enhanced solubility and dissolution rate are developed. Different synthetic strategies (hydrothermal and sol–gel) and aluminum contents are explored to prepare saponites with adjustable stacking. Bumetanide loading is achieved by a solution method. The synthetic route is feasible and scalable. Crystalline and non-crystalline bumetanide co-exist in the hybrids, and the non-crystalline fraction prevails in stacked saponite-based composites. Electrostatic interaction occurs between the negatively charged smectite layers and the protonated anilinic group, and the molecules accommodate within the interlayer spacing of stacked saponites. Compared with bumetanide, all the composites improve water solubility, wettability, and dissolution rate in simulated gastric conditions (pH 1.2 and 4.5) and deionized water. The samples with drug intercalation and a reduced fraction of crystalline bumetanide are the best-performing. The drug release is favoured at pH ≥ 4.5, as electrostatic repulsion occurs between the saponite layers and the carboxylate group of bumetanide. The complete release of the bumetanide dose, not achieved in sufficiently short times for all the fluids considered, is reached in the formulation with appropriate excipients. Bumetanide/saponite composites revealed performant and tuneable systems for bumetanide solubility and dissolution rate enhancement.

## 1. Introduction

The limited aqueous solubility of orally administered Active Pharmaceutical Ingredients (APIs) is a critical factor, as it leads to poor absorption and reduced bioavailability and therefore diminished therapeutic effects. The pharmaceutical industry must explore and develop effective strategies to address this issue, as about 40% of new drugs are lipophilic and fail to reach the required dose due to their low water solubility. Various methods have been developed to enhance the low aqueous solubility and dissolution rate of drugs: particle size reduction and nanonization, cosolvency, pH adjustment, sonocrystallization, solid dispersion, spray drying, and inclusion complexation are just a few examples [1]. In addition, solid multi-component systems including pharmaceutical molecules, such as co-crystals, salts, and co-amorphous systems, have been successfully prepared to modify the physicochemical and pharmacokinetic properties of APIs with limited water solubility [2,3,4,5]. Another appealing approach uses naturally occurring clays to build performant drug delivery systems [6]. Used since ancient times for pharmacological applications and classified as drugs during the Renaissance, clay minerals are now commonly applied as additives in the pharmaceutical industry. Recently, they have proved to be suitable carriers for drugs. In particular, clays are used to delay and/or target drug release, to improve drug dissolution or even to increase drug stability and simultaneously modify drug delivery patterns. Table 1 reports some examples of drug/clay systems recently investigated, including phyllosilicates. The papers thoroughly discuss the drug’s loading mechanism and drug/clay interactions, and the developed systems are performant for controlled drug release. Typically, the studies are carried out by loading the chosen drug onto a clay, natural or synthetic, with a specific composition, structure, and morphology. In addition, it would also be of interest to study how the loading/release mechanism and dissolution behavior of the drug may be tuned by modifying the structural, morphological, and compositional properties of the clay.

**Table 1:** Drug/clay systems investigated for the intercalation of medicinal molecules.

Drug	Clay	Layer Type	Application	References
Praziquantel	Montmorillonite	2:1 layered dioctahedral	Increased dissolution rate and amount of drug dissolved	[7,8]
	Sepiolite	2:1 fibrous trioctahedral		
Olanzapine	Talc-like phyllosilicate	2:1 layered trioctahedral	Increased dissolution rate	[9]
Carvedilol	Halloysite nanotubes	1:1 dioctahedral	Increased water solubility and dissolution rate	[10]
Riparin-A	Laponite	2:1 layered trioctahedral	Increased water solubility and dissolution rate	[11]
Meloxicam	Halloysite nanotubes	1:1 dioctahedral	Increased water solubility and dissolution rate	[12]
Perphenazine	Laponite	2:1 layered trioctahedral	Increased water solubility and dissolution rate	[13]
	Saponite	2:1 layered trioctahedral		
	Halloysite	1:1 dioctahedral		
Quinine·HCl·2H_2_O	Saponite	2:1 layered trioctahedral	Controlled oral drug release	[14]
Nicotine	Magnesium Alumino Silicate (Montmorillonite + Saponite)	2:1 layered (dioctahedral + trioctahedral)	Reduced evaporation and sustained release	[15]
Donepezil	Montmorillonite	2:1 layered dioctahedral	Enhanced release rate	[16]
	Saponite	2:1 layered trioctahedral		
	Laponite	2:1 layered trioctahedral		

**Figure 1:**
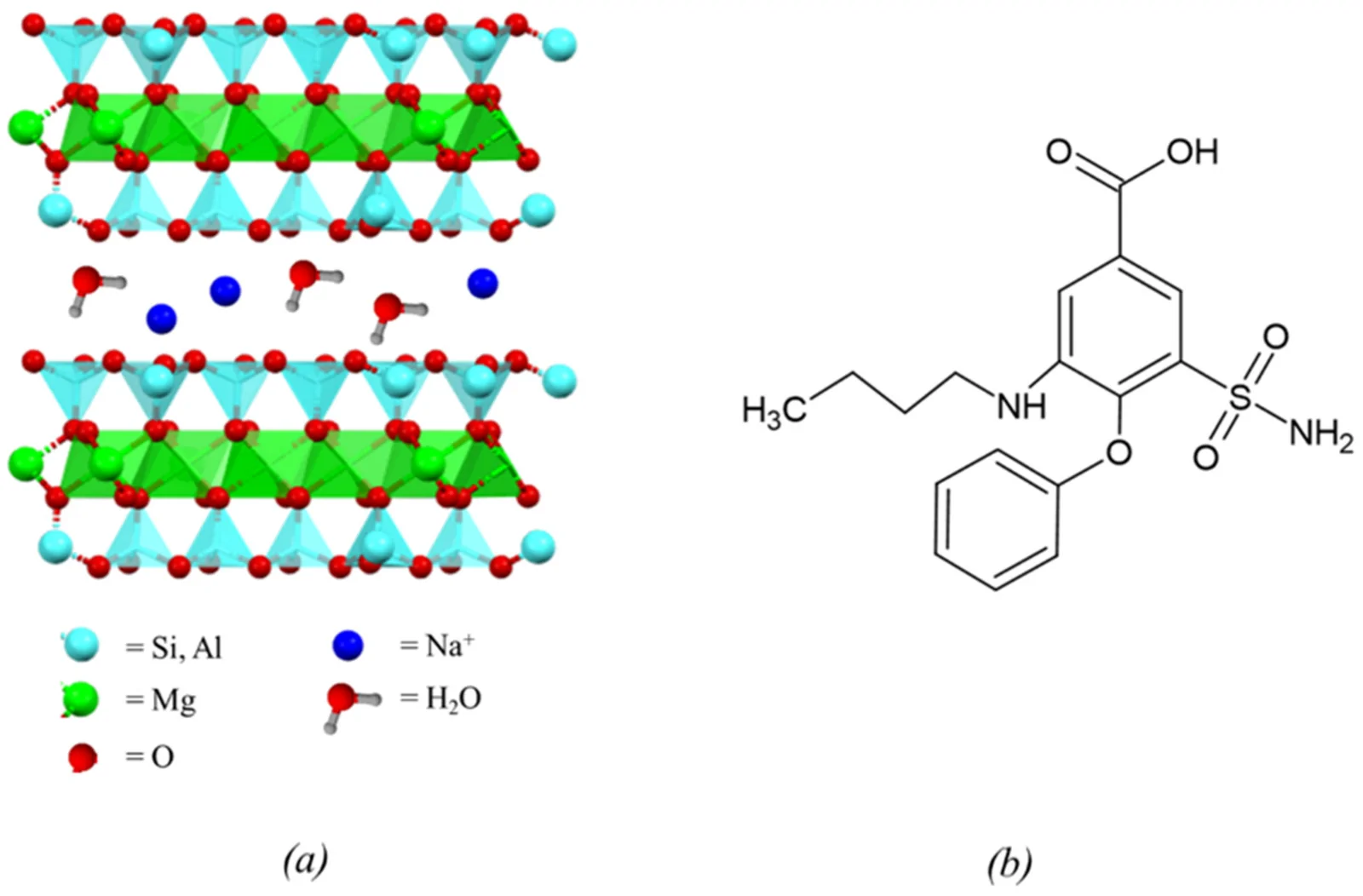
Structure of (**a**) saponite (SAP) and (**b**) bumetanide (BUM), drawn with the CCDC Mercury 4.0 software [31] and ACD/Chemsketch 2.2 [32], respectively.

Saponite is a clay mineral belonging to the smectite group with the chemical formula M_x/z_^z+^[Mg_6_][Si_8−x_Al_x_]O_20_(OH)_4_⋅nH_2_O (0.4 ≤ *x* ≤ 1.2) [17]. The layered structure (Figure 1) consists of one octahedral MgO_6_ sheet sandwiched between two tetrahedral SiO_4_ ones. The M^z+^ cations occupy the interlayer space and balance the layer’s negative charge induced by the substitution of Al^3+^ for Si^4+^. The M^z+^ cations are readily exchanged with inorganic or organic ones, which can be accommodated within the interlayer space because of the swelling behavior of the layered structure. Also, saponite is non-toxic and thermally stable. Although natural saponite is abundant in Earth’s crust and economically attractive, it exhibits some disadvantages compared to its synthetic counterpart. The origin of extraction, the genesis process, and the contaminants present in the deposits introduce variability in chemical composition, impurities, crystallographic defects, and textural properties, all crucial issues that limit its potential use [17,18]. Synthetic saponites represent a valuable alternative for preparing impurity-free clays with different octahedral and tetrahedral metals and tunable composition [19]. The reported features make saponite an appealing mineral for various applications [20], including modulation of drug release [21].

Bumetanide (3-n-butylamino-4-phenoxy-5-sulfamoyl-benzoic acid), with the chemical formula C_17_H_20_N_2_O_5_S and molecular structure shown in Figure 1, is an efficient diuretic for the treatment of edema caused by congestive heart failure, hepatic and renal diseases, and moderate hypertension. It acts in the thick ascending limb of the Henle loop and causes a pronounced urinary excretion of Na^+^, Cl^−^, and other electrolytes, demonstrating to be even 40 times more effective than the well-known furosemide [22,23,24,25].

Bumetanide is characterized by low water solubility and high permeability (BCS Class II drug). It has three ionization constants, 1.44, 3.74, and 10.37, attributed to the anilinic, carboxylic, and sulfonamidic groups, respectively [26]. The solubility in water is pH-dependent and decreases at lower pH. Bumetanide is instead soluble in ethanol, dimethyl-formamide, and dimethyl sulfoxide. The limited aqueous solubility leads to low dissolution rates, and various strategies have been explored to enhance bioavailability, such as the formation of salts and co-crystals [27,28,29] and the design of hydroxyapatite/bumetanide hybrid systems [30].

In the present work, we synthesize saponite/bumetanide hybrid systems and investigate their capability to enhance the drug dissolution rate. To the best of our knowledge, the developed composites have not been previously investigated. In particular, we investigate the influence of the host layer stacking degree on the dissolution behavior of clay/drug systems. To this end, saponites with different degrees of layer stacking and with different aluminum contents (x = 0.6 and 1.2 in the chemical formula) were synthesized through distinct routes and used to load bumetanide. The selected approaches for saponite synthesis, namely hydrothermal and sol–gel methods, are well-established and scalable, enabling the production of highly pure materials with tunable morphology. The bumetanide loading is obtained by a feasible solution method. The selected route to synthesize bumetanide/saponite hybrids may overcome some drawbacks of co-crystals (instability during dissolution, transformation into less-soluble forms, limited scale-up, need for coformer toxicity screening, possible sensitivity to moisture) and hydroxyapatite/drug systems (difficult control of nanoparticle size/morphology, particle agglomeration). The saponites and the saponite/bumetanide hybrid systems are thoroughly characterized from both a physicochemical and pharmaceutical point of view. Finally, the study provides a mechanism for saponite/bumetanide interaction and release and correlates the structural and chemical features of the clay and the amount of the loaded drug with the dissolution behavior of the investigated composites.

## 2. Results and Discussion

Table 2 reports the list of the investigated samples and the alphanumeric codes used.

The effective aluminum content x of the saponite samples, determined by Energy-Dispersive X-ray Spectroscopy (EDS) analysis, is 1.26, 0.62, and 1.04 for SAP-H12, SAP-H06, and SAP-SG, respectively, which fairly match the theoretical aluminum content (Table 2).

### 2.1. Powder X-Ray Diffraction (PXRD)

**Figure 2:**
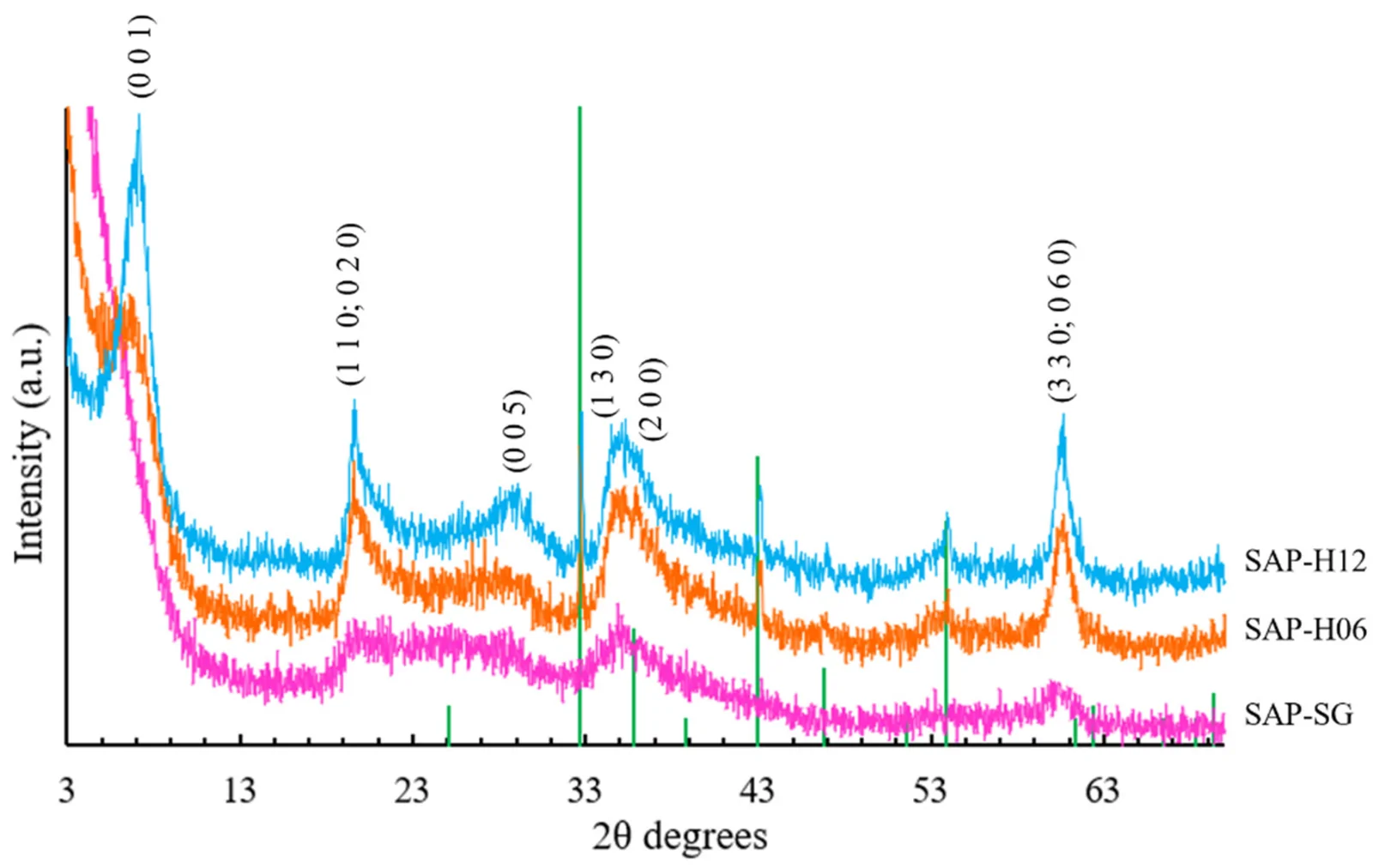
PXRD patterns of saponite samples. Green lines: magnesite impurity of hydrothermal saponites (JCPDS card 08-0479).

Figure 2 shows the diffraction patterns of the saponite samples. Details on the peak position of the (001) reflection, impurity phase amounts, and crystallite size are reported in Table S1. The main peaks of saponite are detected in all the samples, in accordance with the literature data [33,34] and JCPDS database (JCPDS card 12-0157). However, differences occur concerning impurity phases, layer stacking, and crystallite size. A progressive decrease in the intensity of the (001) reflection is observed from the hydrothermal to the sol–gel saponites, suggesting that the synthetic route affects the degree of layer stacking. Hydrothermal synthesis gives a stacked structure, more pronounced in SAP-H12 with a higher aluminum content (x = 1.26 vs. x = 0.62). The stacking is instead negligible for the sol–gel sample, as expected for this synthetic approach [35]. The crystallite size, evaluated by applying the Scherrer equation to the broadening of the peak at about 60.7°, is nanometric and smaller for the sol–gel sample (about 11 nm for hydrothermal samples vs. 5.0 nm for the sol–gel one). A small amount of magnesite impurity (<4% *w*/*w*) is also detected in the hydrothermal samples and is quantified by the ratio of the integrated intensity of magnesite peaks to the total intensity (magnesite plus saponite). The magnesite formation is consistent with the use of the buffer solution (NaHCO_3_ in NaOH, pH = 13) in the synthesis. The saponite sample synthesized via sol–gel is impurity-free.

**Figure 3:**
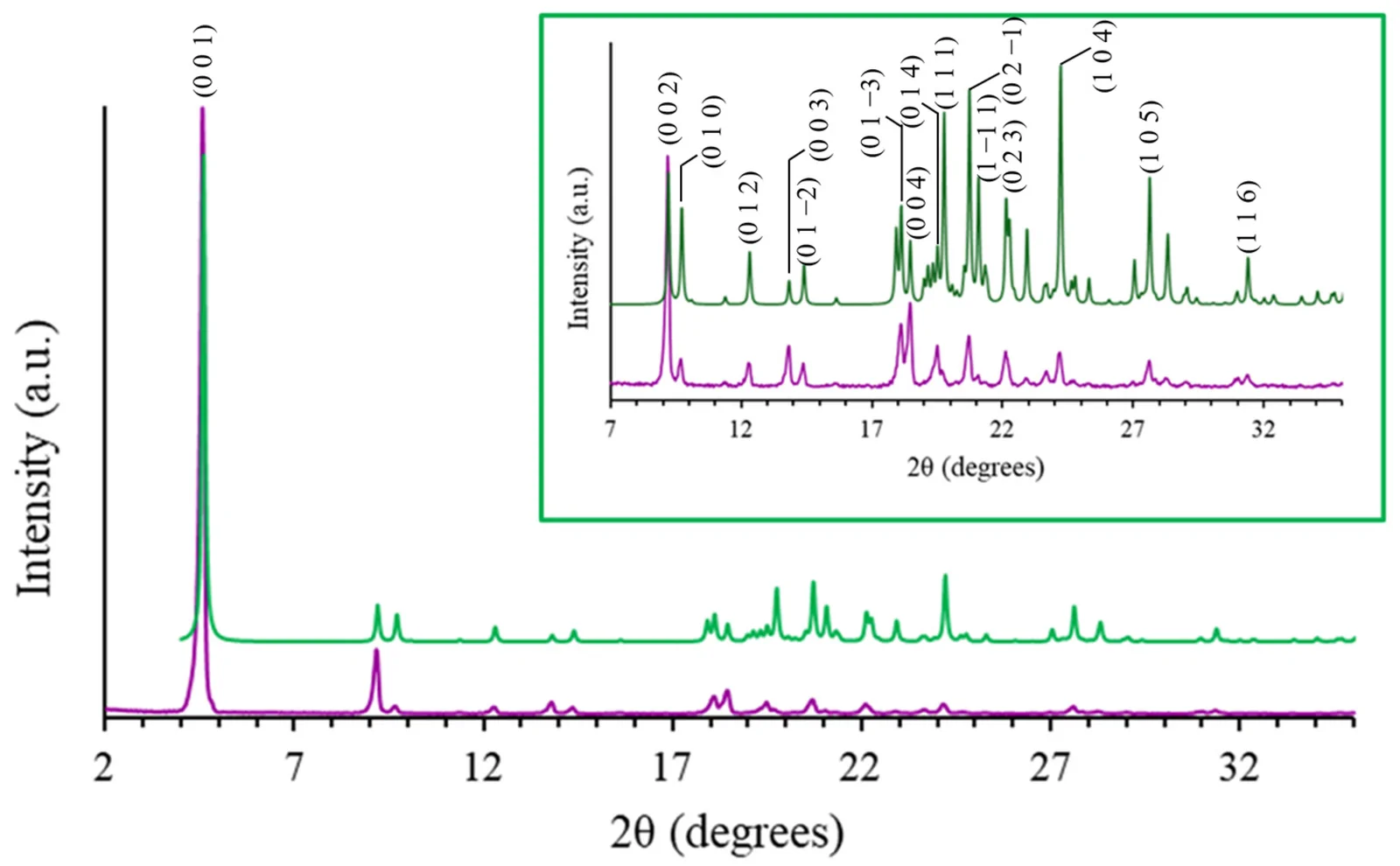
PXRD pattern of the bumetanide sample (violet) and calculated pattern of the triclinic crystal structure (green). Inset: magnification of the 7–35° angular range.

Figure 3 shows the diffraction pattern of the bumetanide active principle. The sample is crystalline. Bumetanide crystallizes into two polymorphic forms, with comparable energies and molecule conformations: (i) monoclinic, *P*2_1_/*c* Space Group, *a* = 5.3952(13) Å, *b* = 18.206(4) Å, *c* = 18,119(4) Å, *β* = 98.222(2)°, V = 1761.4(7) Å^3^, Z = 4 [36]; (ii) triclinic, *P*-1 Space Group, *a* = 5.00168(4) Å, *b* = 9.22649(3) Å, *c* = 19.59924(14) Å, α = 80.7941(5)°, *β* = 82.8401(7)°, *γ* = 86.8148(7)°, V = 885,268(9) Å^3^, Z = 2 [37].

The diffraction data of the bumetanide sample are exhaustively explained by the triclinic polymorph (calculated pattern in Figure 3) and match the patterns reported in the literature [28,30,38]. Table S2 summarizes the two-theta positions, intensities, *d*-spacing, and Miller indices of the main peaks of triclinic bumetanide. Figure S1 displays the triclinic crystal structure.

**Figure 4:**
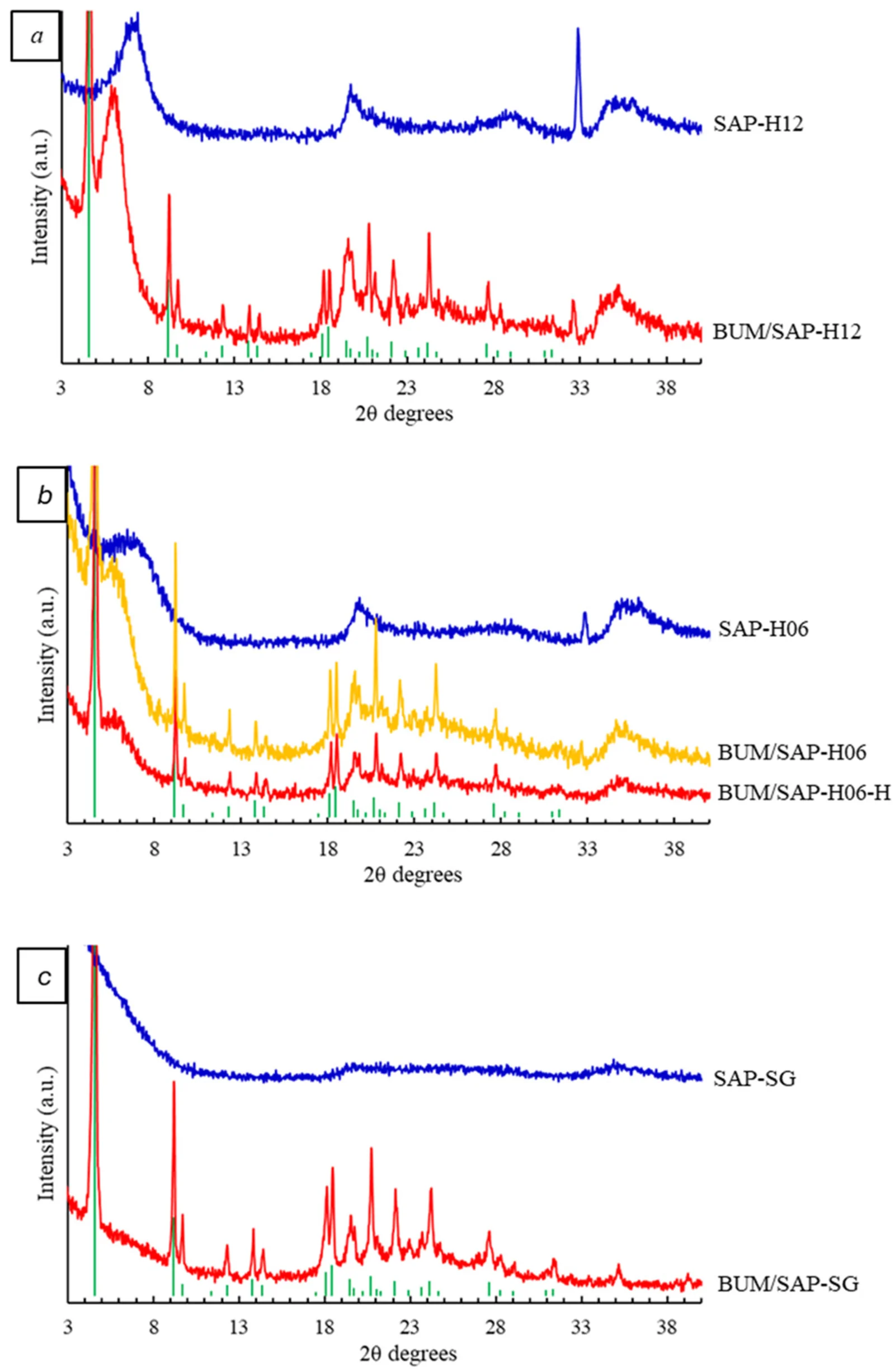
Diffraction patterns of pristine saponites and drug/saponite systems: (**a**) SAP-H12 and BUM/SAPH12; (**b**) SAP-H06, BUM/SAP-H06, and BUM/SAP-H06-H; (**c**) SAP-SG and BUM/SAP-SG. The position and intensity of the bumetanide peaks are also shown (green bars).

Figure 4 displays the diffraction patterns of the bumetanide/saponite composites and of the pristine clays for comparison. The loading of bumetanide on hydrothermal saponites (i.e., saponites with stacking of the aluminosilicate layers witnessed by the intensity of the (001) basal plane) causes a shift in the two-theta position of the (001) reflection towards lower angles. The *d*_001_ interplanar spacing increases from 12.49 Å (SAP-H12 sample) to 14.83 Å for BUM/SAP-H12 and from 12.46 Å (SAP-H06 sample) to 15.66 Å and 14.83 Å for BUM/SAP-H06 and BUM/SAP-H06-H, respectively. The interlayer spacings, obtained by subtracting the thickness of the TOT smectitic layers (about 9 Å) from the *d*_001_ value of the composites, are 5.83, 6.66, and 5.83 Å, suitable to accommodate the bumetanide molecules, as suggested by the computational modeling results discussed in Section 2.2. The protonated anilinic group can interact with the negatively charged aluminosilicate layers. The other saponite peaks are also detected. In addition, several sharp peaks pertinent to bumetanide in the triclinic form are present, suggesting the co-existence of crystalline and intercalated bumetanide in the samples. In the case of the bumetanide/sol–gel saponite composite, the drug interacts with a non-stacked clay structure and crystallizes in the triclinic structure. The PXRD results suggest the loading of bumetanide onto the saponites. In all the composites, crystalline bumetanide is also detected.

### 2.2. Computational Modeling

The optimized structure of protonated bumetanide (Figure S2) corresponding to the global minimum on the potential energy surface exhibits approximate dimensions of 14.9 × 13.2 × 8.4 Å (taking into account of the contribution of VdW radii), which appear incompatible with intercalation within the clay lamellar units. As shown in Figure S3a, this relatively bulky conformation mainly arises from the orientation of the phenyl ether aromatic ring with respect to the rest of the molecule. However, this geometry represents only one of several accessible conformations. A potential energy surface scan of the rotation around the C–O bond connecting the phenyl ether moiety (highlighted in Figure S2b) revealed that the compound can assume alternative conformations through modest-energy rearrangements, with rotational barriers in the range of 1.4–2.3 kcal mol^−1^. These conformers are significantly more compact (an example is displayed in Figure S3b) and exhibit molecular thicknesses of approximately 6.7 Å, compatible with accommodation within the clay interlayer region.

### 2.3. Morphological Investigation

**Figure 5:**
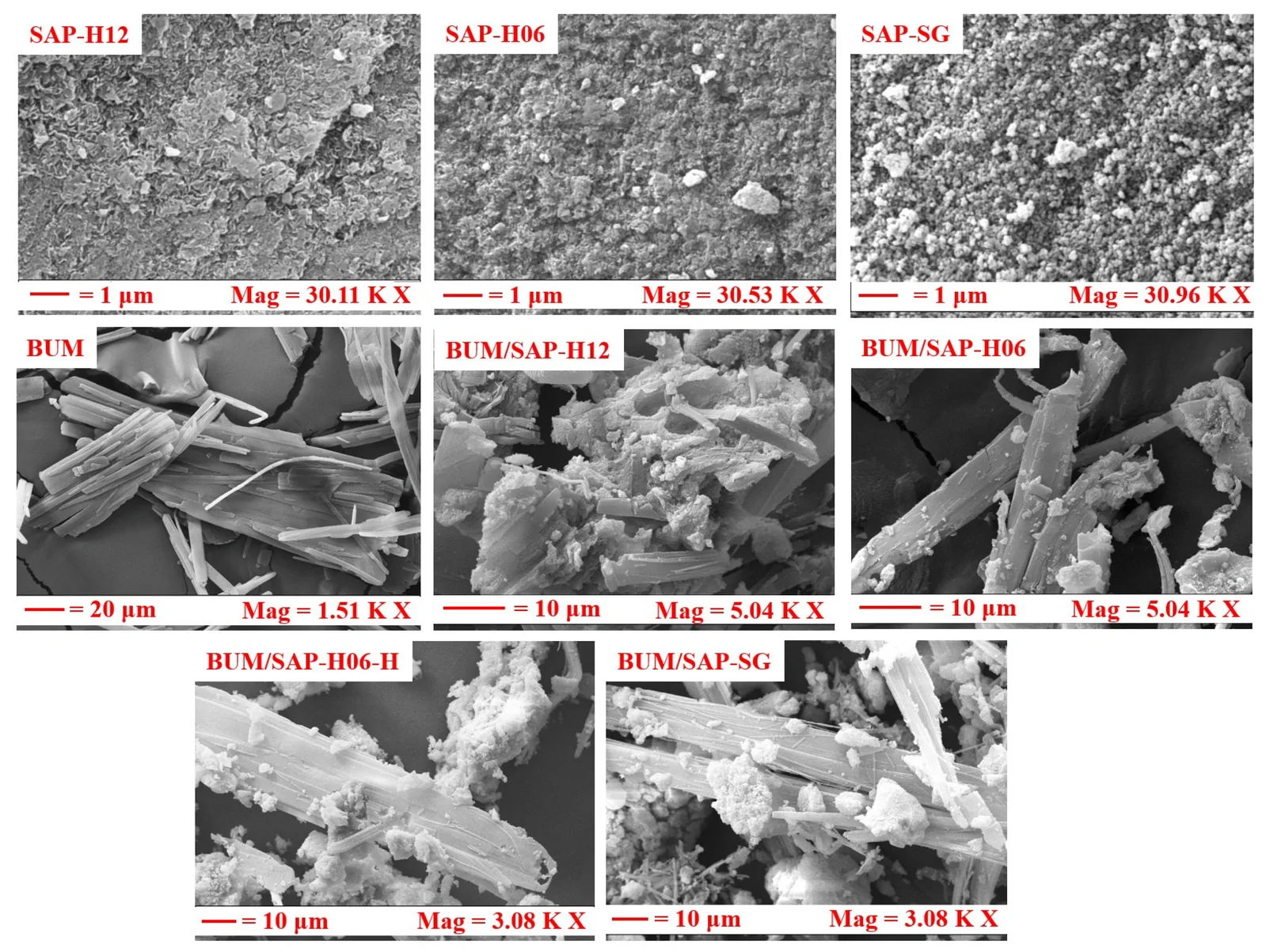
SEM images of saponites, bumetanide, and bumetanide/saponite hybrids.

The multiphase nature of the hybrids (bumetanide–clay system and crystalline bumetanide) is also confirmed by Scanning Electron Microscopy (SEM) investigation. Figure 5 shows the SEM images of the saponites, bumetanide, and bumetanide-clay composites. The saponite samples via hydrothermal synthesis exhibit aggregates of pseudo-lamellar micrometric particles, resembling the layered nature of the mineral; the lamellar morphology is more evident in BUM/SAP-H12, which displays an intense (001) reflection in the diffraction pattern. A different morphology based on agglomerates of rounded nanometric particles is instead observed for the sol–gel sample; this is consistent with the PXRD results, which show negligible stacking. Bumetanide consists of aggregates of needle-shaped particles with a micrometric diameter and lengths of several hundred micrometres. The SEM images of the composites show both saponite aggregates and bumetanide particles. This result is consistent with the crystalline drug component detected by PXRD in all the investigated hybrid systems.

### 2.4. Bumetanide Content

**Figure 6:**
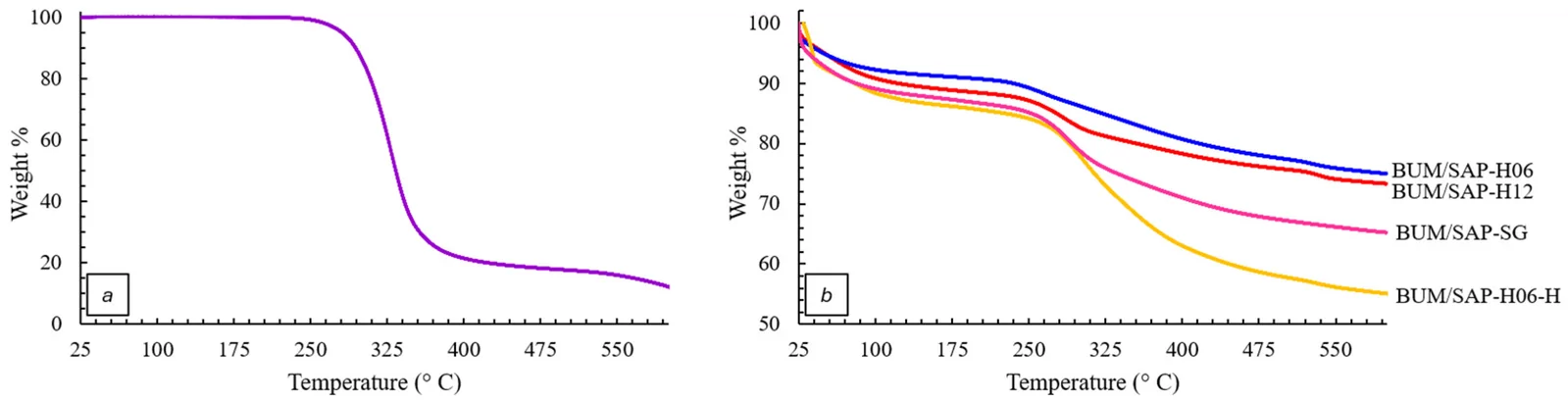
TGA curves of (**a**) BUM and (**b**) BUM/SAP hybrids.

The bumetanide loaded in the hybrid samples is quantified by UV–Vis spectroscopy and thermogravimetric analysis. The UV–Vis results are reported in Table 3. The drug loading is quite good from a pharmaceutical viewpoint. It is 12.0% *w*/*w* for BUM/SAP-H06, 11.5% *w*/*w* for BUM/SAP-H12, and 17.8% *w*/*w* for BUM/SAP-SG. As expected, a higher content, 29.1% *w*/*w*, is observed for BUM/SAP-H06-H because it is synthesized with twice the amount of BUM compared to the other samples. The drug amount, expressed as the percentage of the theoretical bumetanide value, is 60%, 57.5%, 89% and 72.75% for BUM/SAP-H06, BUM/SAP-H12, BUM/SAP-SG and BUM/SAP-H06-H, respectively, confirming that the selected loading method is effective. The sol–gel sample exhibits the highest loading. The thermogravimetric analysis (TGA) curves of the saponite samples are shown in Figure S4. They exhibit a significant mass loss in the 25–170 °C temperature range, attributed to the release of physisorbed water. As regards SAP-H06, an additional net mass loss is detected at about 180, then a gradual mass loss occurs up to 475 °C, followed by another net mass loss over 500 °C. The mass is almost constant up to 500 °C for SAP-H12, whereas a small progressive mass loss occurs for SAP-SG. Independently of the investigated saponite, the mass loss in the 170–450 °C temperature range, differently pronounced depending on the sample, is attributed to the removal of the remaining strongly bound interlayer water molecules. Finally, the mass release at higher temperature is due to the dehydroxylation of the saponite structure. Figure 6 displays the TG curves of bumetanide and bumetanide/clay samples. Bumetanide is stable up to about 200 °C, then a mass loss occurs in the 200–450 °C temperature range due to drug decomposition. The TG curves of the bumetanide/clay samples resemble the mass losses detected in the clays (25–200 °C) and the drug (200–450 °C). Using the mass losses in the 200–450 °C temperature range of the pristine clays, of bumetanide, and of the bumetanide/clay composites (Table S3), the drug content of the hybrid systems is evaluated and reported in Table 3. The weight percentages match those obtained by UV–Vis spectroscopy.

**Figure 7:**
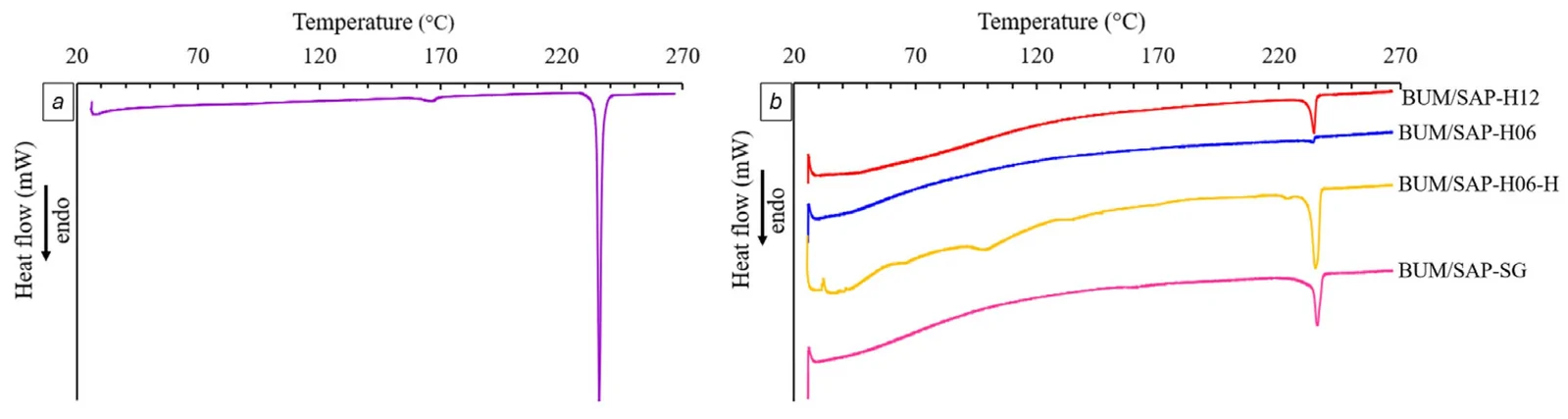
DSC curves of (**a**) bumetanide and (**b**) bumetanide/clay composites.

The Differential Scanning Calorimetry (DSC) curves of the saponite samples (Figure S5) exhibit only a broad endothermic peak in the 25–170 °C temperature range, attributed to the release of adsorbed water. The DSC curves of bumetanide and bumetanide/clay hybrids are shown in Figure 7. Pure bumetanide exhibits a polymorphic transition at about 160 °C, followed by a net and sharp endothermic peak at 234.6 °C (ΔH = 123.3 J/g) due to the melting process [28]. The bumetanide/clay composites show the melting peak of bumetanide at about 235 °C, according to the presence of crystalline bumetanide in the samples, as demonstrated by PXRD. The T_onset_, the enthalpy variation in the melting process, and the weight percentage of the crystalline bumetanide in the hybrid samples are reported in Table S4. The percentage of non-crystalline drug (weight percentage of non-crystalline bumetanide over the total bumetanide loaded in each sample and determined by UV–Vis quantification) is calculated and reported in Table 3. The results confirm that the bumetanide/clay hybrids contain both crystalline and non-crystalline bumetanide. Higher percentages of non-crystalline drug are detected in the hybrids synthesized with 20% *w*/*w* of drug loaded onto hydrothermal saponites. In this case, bumetanide can be accommodated within the aluminosilicate layers, as demonstrated by PXRD and computational modeling. The use of a higher amount of bumetanide in the synthesis (40% *w*/*w*: BUM/SAP-H06-H sample) does not increase the drug content in the interlayer spacing, as bumetanide preferentially segregates in its crystalline form. In the case of the BUM/SAP-SG sample, the layer stacking of saponite is negligible, and bumetanide mainly segregates as a separate phase in the crystalline form.

### 2.5. Attenuated Total Reflectance–Fourier Transform Infrared Spectroscopy (ATR-FTIR)

The acronym FT-IR, meaning ATR-FTIR measurements, will be used hereafter for shortness. Figure S6 shows the FT-IR spectra of the saponite samples. As expected, they exhibit the same signals. The Mg-OH stretching and bending are attributed to the bands at 3680 and about 650 cm^−1^, respectively. The Mg-Al-OH peak is around 3616 cm^−1^. The broad band at about 3400 cm^−1^ and the sharp one at about 1630 cm^−1^ refer to the HO-H stretching and bending, respectively. Finally, in the 960–980 cm^−1^ and 800–820 cm^−1^ regions, the Si-O-Si and Si-O-Al stretching modes are detected. The Si-O-Si band position is influenced by both the isomorphic substitution and the degree of layer stacking. The Al-O bond is longer and weaker than the Si-O bond, thus causing a red shift (964 cm^−1^ for SAP-H12 and 976 cm^−1^ for SAP-H06). The blue shift in the SAP-SG sample (989 cm^−1^) is attributed to the reticular disorder and lack of stacking that move the band to values closer to those of disordered silica [39,40,41]. In the hydrothermal samples, an additional band centred at about 1390 cm^−1^ is detected. It can be attributed to the presence of a small amount of magnesite impurity, as previously discussed in the PXRD results.

**Figure 8:**
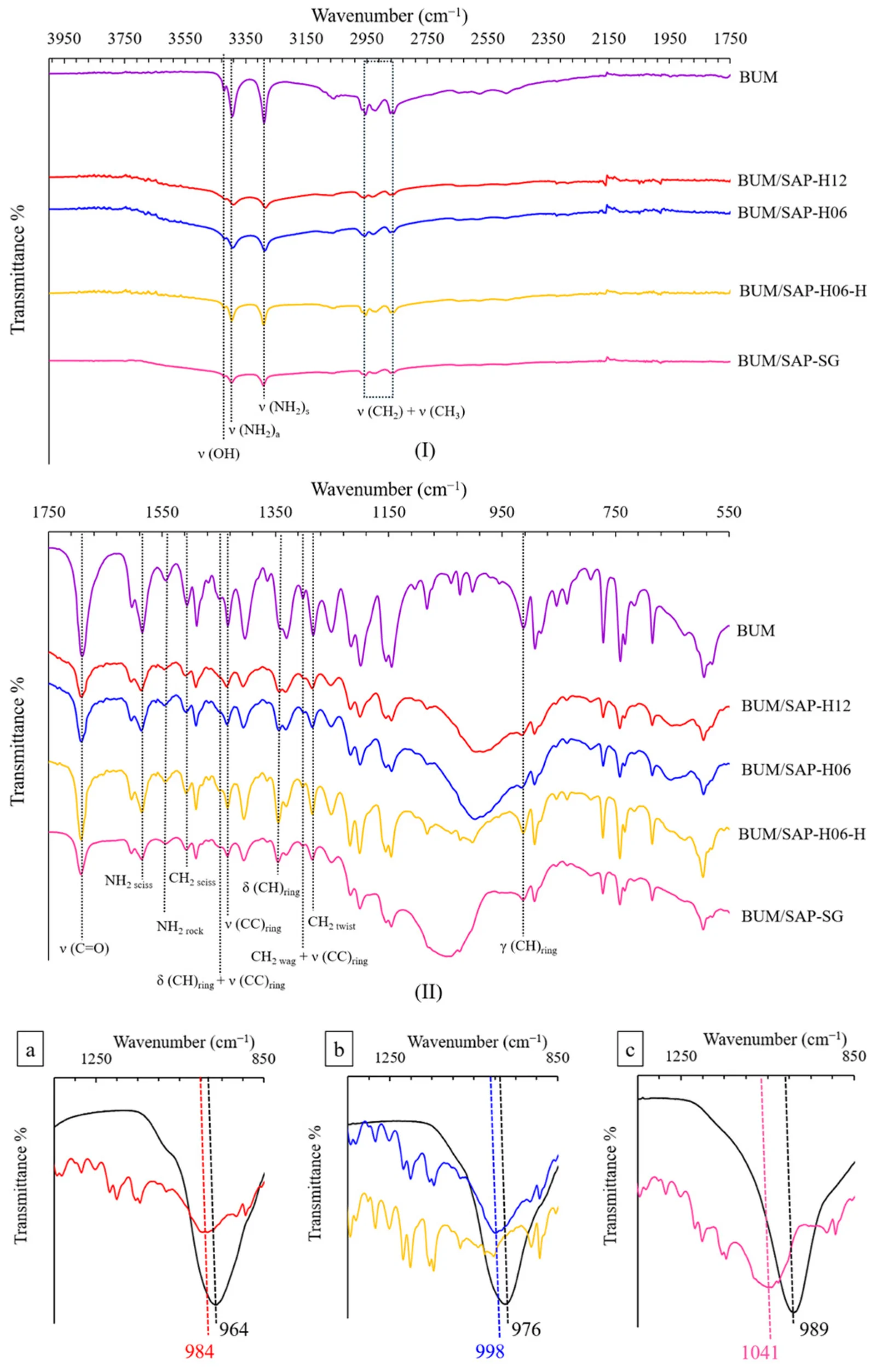
FT-IR spectra of BUM and BUM/SAP hybrids in (**I**) 4000–1750 cm^−1^ region and (**II**) 1750–550 cm^−1^ region. Details of the Si-O-Si stretching band of saponite in (**a**) SAP-H12 and BUM/SAP-H12 (red), (**b**) SAP-H06 and BUM/SAP-H06 and (**c**) SAP-SG and BUM/SAP-SG samples. Saponites: black line.

FT-IR spectra of bumetanide and of the composites are reported in Figure 8. The bumetanide spectrum matches all the assignments reported in the literature [42]. The FT-IR bands of bumetanide are assigned in Table S5. Peak positions and vibrational mode assignments of all the samples are reported in Table 4. The shifts in band positions between pure components and composites are evaluated to evidence possible interactions. A shift in the Si-O-Si stretching mode of the saponite occurs for all the loaded samples (Figure 8a–c). This may be attributed to a perturbation of the local electrostatic environment of the Si-O-Si bond, even if a contribution from the superposition of the bumetanide peaks cannot be ruled out. For the BUM/SAP-SG sample, this phenomenon is more evident: the completely non-stacked structure of SAP-SG provides highly accessible external siloxane surfaces, favoring direct interactions between the protonated aniline groups of bumetanide and surface oxygen atoms, resulting in a stronger variation in the vibrational frequency. The stretching modes of anilinic NH_2_, which is partially protonated under synthetic conditions, shift when interacting with the saponites with a major negative charge (i.e., SAP-H12 and SAP-SG); the corresponding bending modes shift when interacting with stacked structures, whereas no evident shifts occur in the BUM/SAP-SG sample. This suggests a partial intercalation of the drug in the interlayer space. This hypothesis is further supported by the more pronounced shifts in the ring vibrational modes, mainly evident in the BUM/SAP-H06 sample, which has the highest content of non-crystalline bumetanide loaded into the saponite interlayer space. As regards the signals related to the aliphatic lateral chain, the shifts are recorded in the BUM/SAP-H06 and BUM/SAP-H12 hybrids, which are characterized by a lower content of crystalline drug, as demonstrated by DSC analysis. The reported FT-IR results for the drug/hydrothermal saponite hybrids also suggest possible local rearrangement/distortion of the bumetanide molecules to accommodate into the interlayer spacing of the stacked saponite structure.

### 2.6. Solubility

The BUM equilibrium solubility in deionized water at 25 °C is 23.0 ± 1.6 mg/L, while BUM/SAP-H06 shows an almost 8-fold increase to 178.2 ± 9.5 mg/L, and BUM/SAP-SG shows an approximately 4-fold increase from the BUM solubility: 88.9 ± 4.3 mg/L. The obtained values are higher than those obtained for bumetanide−4-aminobenzoic acid molecular co-crystals (1:1 stoichiometric ratio), which exhibit an equilibrium solubility in distilled water of 32.6 ± 1.2 1 mg/L [28], and they are also higher in comparison with a hydroxyapatite/bumetanide system reported in [30], which shows a 40.2 ± 1.3 mg/L water solubility. The trend in the solubility values of the hybrid systems is consistent with the content of crystalline bumetanide.

### 2.7. Wettability

**Figure 9:**
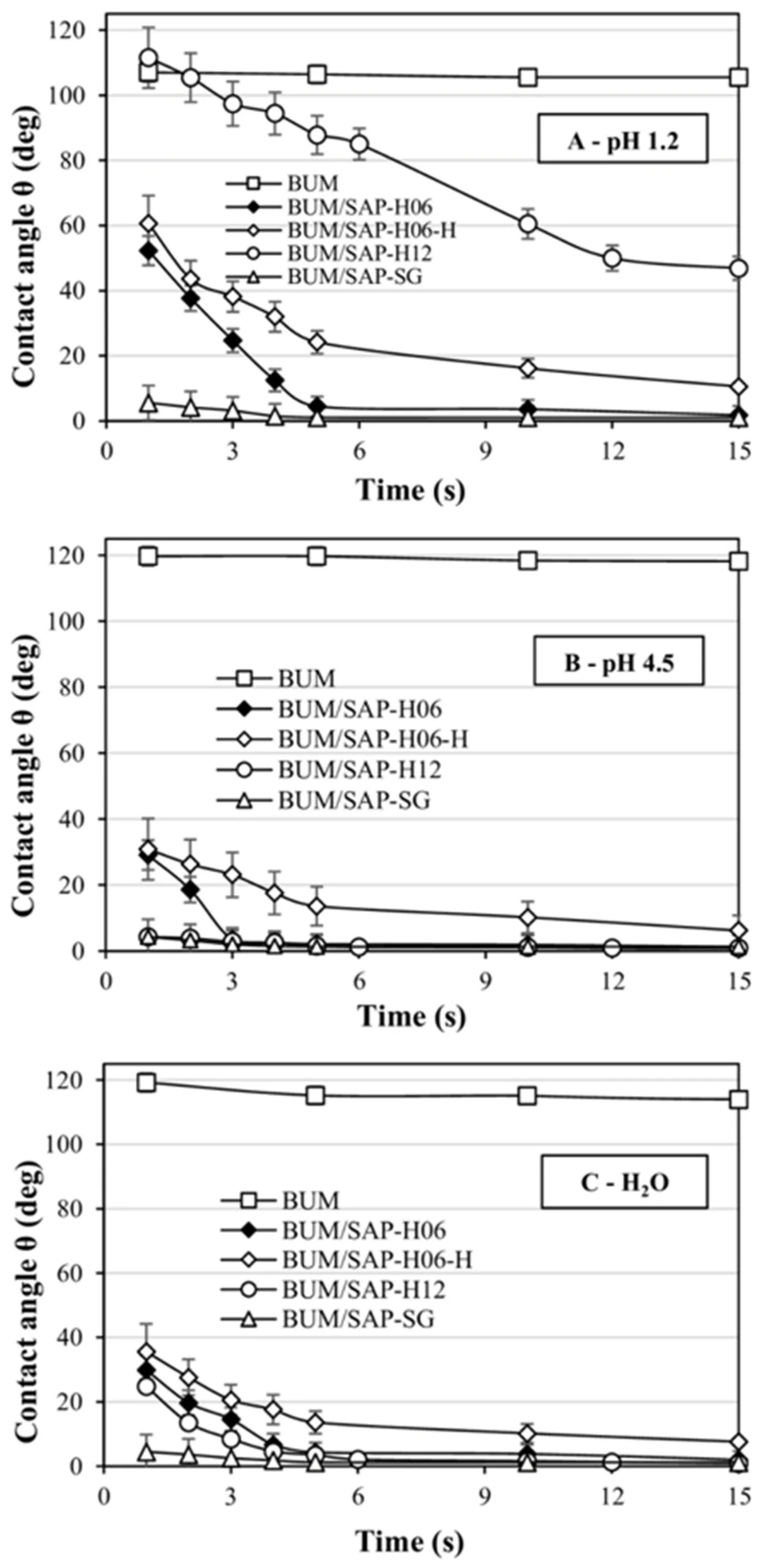
Contact angle measured on BUM and BUM/SAP hybrids.

The contact angle measurements provide evidence of improved wettability in the BUM/SAP samples compared to BUM alone (Figure S7). In all fluids considered, the samples loaded onto saponite show a contact angle much lower than the pure drug, which rapidly decreases to zero within a few seconds (Figure 9). At only pH = 1.2, the BUM/SAP-H12 sample initially shows a contact angle very similar to that of BUM, but, also in this case, the value progressively decreases over time and reaches zero in about 45 s.

### 2.8. Dissolution Tests

**Figure 10:**
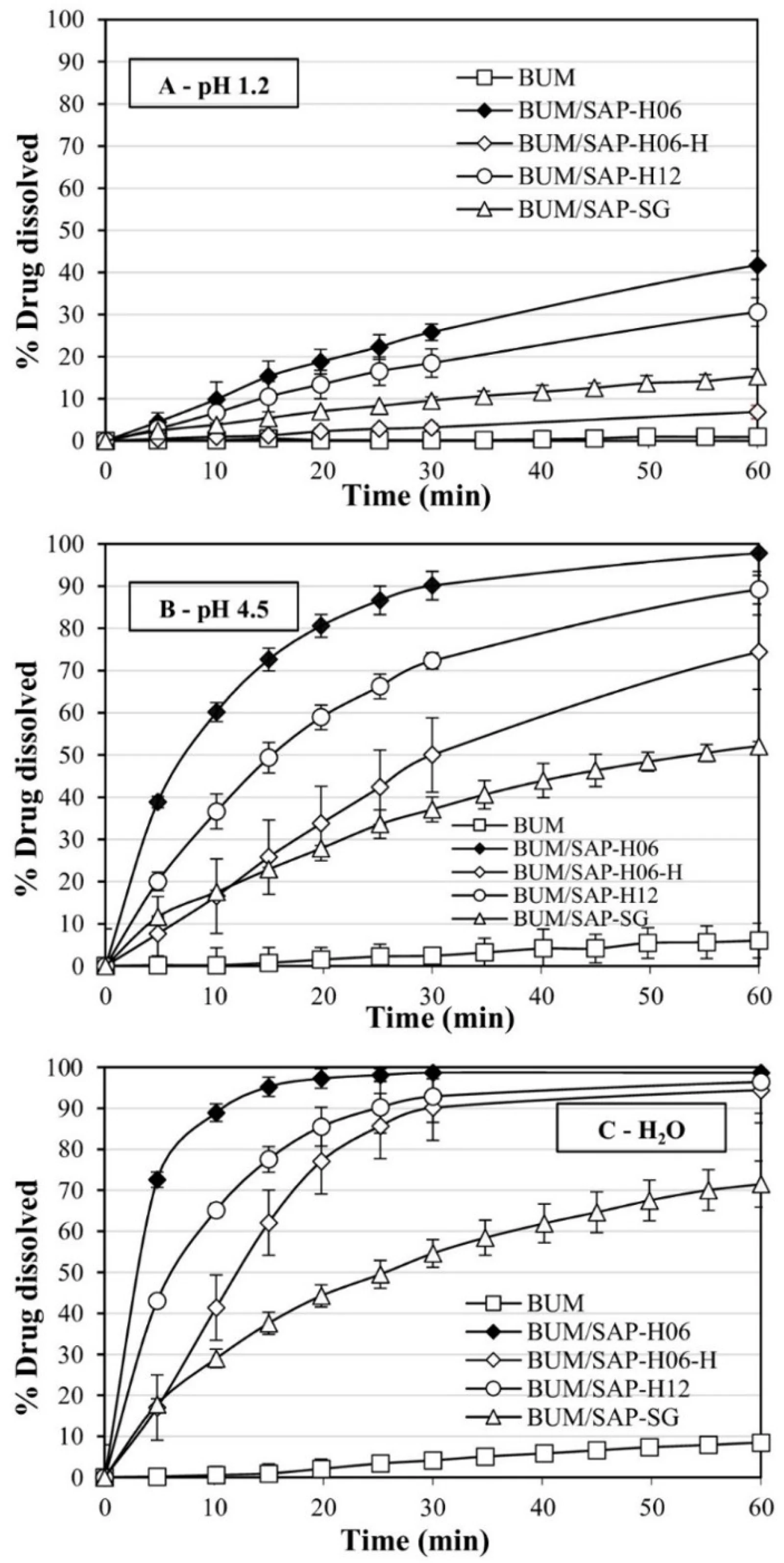
Dissolution profiles of BUM and BUM/SAP systems in the different fluids.

Dissolution tests show a significant increase in the rate of BUM solubilization from all the analyzed compounds (Figure 10). In deionized water (the fluid required by the USP monograph for the routine testing of BUM tablets), all saponite samples show a significant increase in the drug dissolution rate, with the greatest improvement for BUM/SAP-H06, followed by BUM/SAP-H12, BUM/SAP-H06-H, and finally BUM/SAP-SG. Consistent with the physicochemical characterization, samples containing a lower fraction of crystalline drug, likely due to its incorporation within the carrier layers, exhibit higher dissolution rates. This demonstrates a progressive effectiveness of saponite in improving the drug’s dissolution characteristics as a function of its intercalation properties. Compared to the previously investigated hydroxyapatite/bumetanide system [30], the BUM/SAP-H06 sample exhibits worse performance at an acidic pH (1.2), while some enhancement in the dissolution behavior is observed at pH 4.5 and in deionized water. The marked different behavior at pH = 1.2 and 4.5 may be explained by the drug release mechanism, discussed in Section 2.9. At pH = 1.2, actractive electrostatic interactions occur between the protonated anilinic group of the bumetanide molecule and the negatively charged alluminosilicate layers, which inhibit drug release. Indeed, the bumetanide release is facilitated at pH = 4.5, as electrostatic repulsion occurs between the carboxylate groups of the drug and the clay layers.

**Figure 11:**
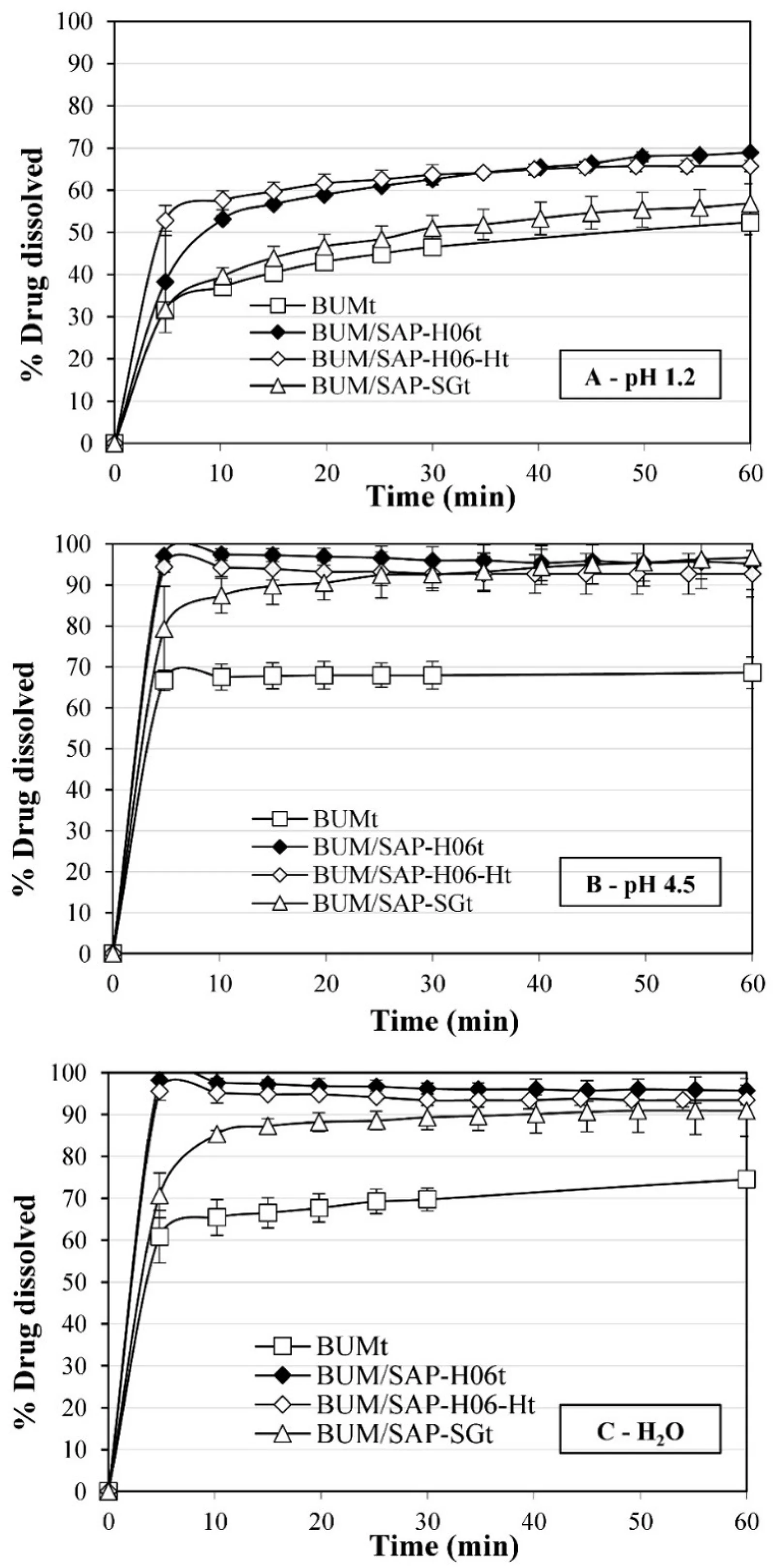
Dissolution profiles of the formulated tablets containing the BUM/SAP systems.

However, in some fluids (in simulated gastric fluid, pH = 1.2, and in the pH = 4.5 buffer, which simulates the gastric environment in the fed state), the dose is not completely released in sufficiently short times to ensure a rapid absorption of the drug, especially in emergency cases when the most immediate therapeutic effect possible is required. For this reason, some compounds were selected for a tablet formulation, BUM/SAP-H06t, BUM/SAP-H06-Ht, and BUM/SAP-SGt, and their dissolution profiles were compared with the same formulation containing pure BUM: BUMt (Figure 11).

Thanks to the use of suitable excipients to further enhance drug solubilization, the dissolution rate of bumetanide is further increased, but only the samples loaded on saponite can guarantee immediate release of the entire dose in less than 10 min in water and in buffer at pH = 4.5. Even at pH = 1.2, a significant improvement in drug availability is observed: approximately 60% of the dose passes into solution in 10 min from the BUM/SAP-H06t sample and in 15 min from BUM/SAP-H06-Ht, while only half of this fraction is released from BUM/SAP-SG and the tablets containing pure bumetanide. In all cases, after this very rapid initial phase, the remainder of the dose is released, although more slowly.

### 2.9. Mechanism of Bumetanide–Saponite Interaction and Release

**Figure 12:**
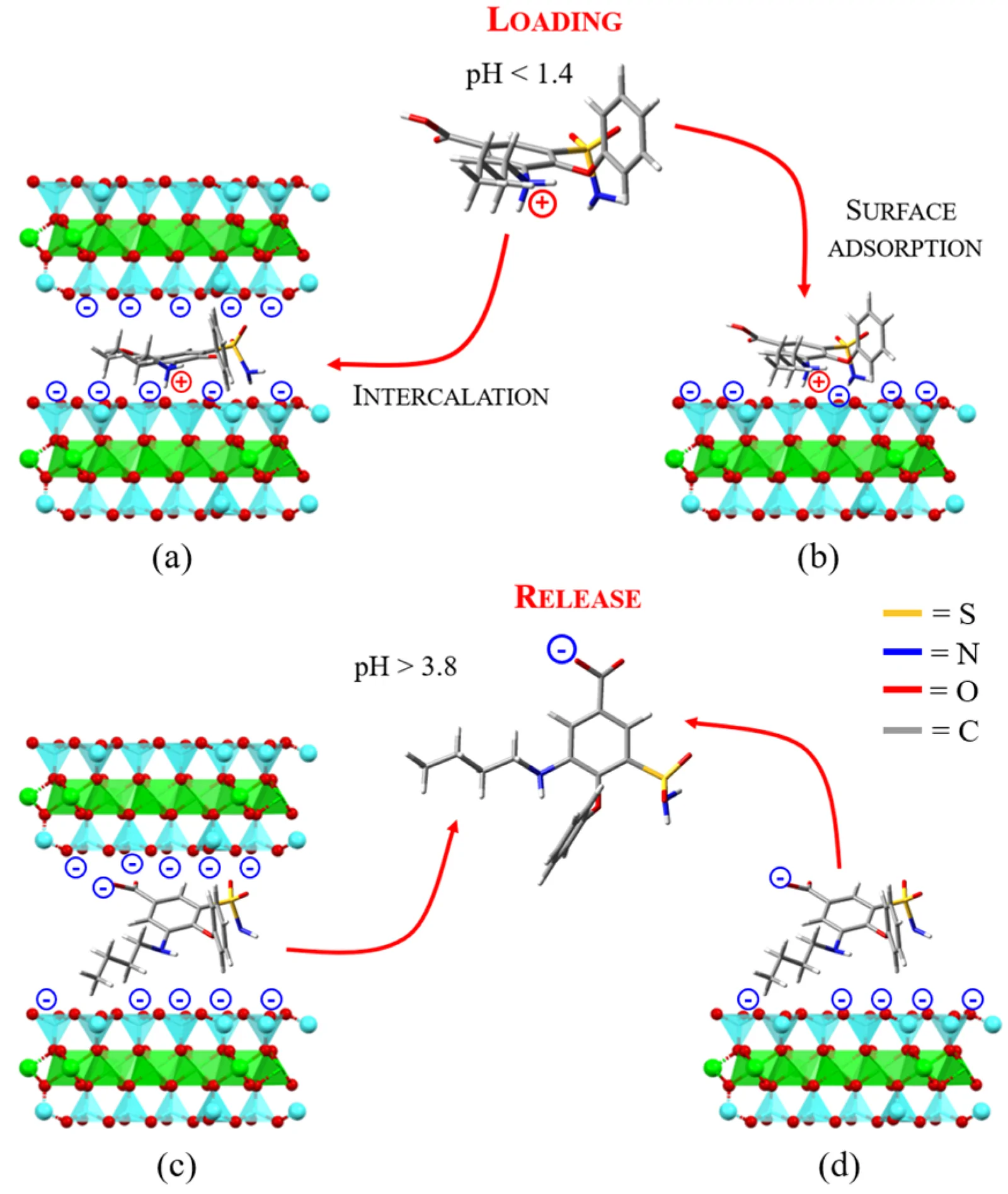
Bumetanide loading mechanism based on the protonated anilinic group and the clay layer attraction in (**a**) stacked and (**b**) non-stacked saponite. Bumetanide release mechanism at pH ≥ 4.5 based on the carboxylate groups and the layer repulsion in (**c**) stacked and (**d**) non-stacked saponite.

The results obtained by PXRD, FT-IR, computational modeling and dissolution tests allow us to elucidate the interaction and release mechanism of bumetanide in the investigated hybrids. These systems are quite complex, as the drug is present in both crystalline and non-crystalline form. Furthermore, the relative abundance of the two forms depends on the structural properties of the host. In the case of stacked saponites obtained by hydrothermal synthesis (BUM/SAP-H12, BUM/SAP-H06 samples), the non-crystalline component prevails. The bumetanide molecules intercalate within the clay interlayer region and exhibit a compact conformation with a molecular thickness compatible with accommodation within the clay interlayer region. Electrostatic interaction occurs between the negatively charged aluminosilicate layers of the clay and the protonated anilinic group of the drug, as revealed by the FT-IR shifts in the Si-O-Si bond and in the anilinic stretching and bending modes. The shifts in the ring and the aliphatic lateral chain vibrational modes of the drug are consistent with possible molecule rearrangements required to enter the interlayer space of saponite. Figure 12a displays the intercalation mechanism in the layered structure. In the case of non-stacked saponite synthesized by the sol–gel route, electrostatic interactions again occur between the negatively charged aluminosilicate layers of the clay and the protonated anilinic group of the drug molecules. The FT-IR shifts of the Si-O-Si bond are more pronounced than in the hydrothermal hybrids, consistent with the non-stacked structure of SAP-SG, which provides highly accessible external siloxane surfaces. No shifts in the ring vibrational modes and in those of the aliphatic lateral chain of the drug are detected, as the drug molecules are not constrained within the saponite layers. Figure 12b displays the clay–drug mechanism in the non-stacked structure of saponite. To explain the bumetanide release, both crystalline and non-crystalline saponite have to be considered. In addition, based on the dissolution behavior of pure bumetanide (crystalline), we do not expect a relevant role of the crystalline component. Independently of the pH, drug release is strictly related to the non-crystalline component of the hybrids, i.e., to the drug interacting with the aluminosilicate layers of the saponites. Hybrids with higher amounts of the non-crystalline component perform better. Notably, a significant increase in dissolution rate and released dose is achieved at pH = 4.5 compared to pH = 1.2. In the pH = 4.5 buffer, which simulates the gastric environment in the fed state, both the anilinic and carboxylic groups are deprotonated (ionization constants of anilinic and carboxylic groups: 1.44 and 3.74, respectively). The electrostatic repulsion between the negatively charged aluminosilicate layers and the carboxylate group could act as a driving force for the release of the bumetanide molecules at pH ≥ 4.5. The release mechanism is shown in Figure 12c,d.

## 3. Materials and Methods

### 3.1. Materials

All the chemicals employed were of reagent-grade quality or higher. Bumetanide was kindly donated by Fidia Farmaceutici S.p.A. (Paderno Dugnano, Italy). Na_2_SiO_3_ solution (25.9% SiO_2_) was purchased from VWR International s.r.l. (Milan, Italy). Al(NO_3_)_3_·9H_2_O, NaOH, Mg(NO_3_)_2_·6H_2_O, urea, EtOH and NaHCO_3_ were purchased from the Merck Company (Milan, Italy). For the tablet production, the following excipients were used: microcrystalline cellulose NF, Ph.Eur., JP (Avicel^®^ PH101, FMC BioPolymer, Brussels, Belgium), crosslinked polyvinyl pyrrolidone (Polyplasdone^®^ XL, GAF Corporation, New York, NY, USA), sodium hydrogen carbonate (Carlo Erba, Milan, Italy), Polyoxyethylene-polyoxypropylene triblock copolymer, Poloxamer 407 (Lutrol^®^ F127 MICRO, BASF, Ludwigshafen, Germany), sodium lauryl sulfate (Carlo Erba, Milan, Italy), magnesium stearate (Carlo Erba, Milan, Italy), and colloidal silicon dioxide (Syloid^®^ 72FP, Grace, Worms, Germany). All other excipients were of pharmaceutical/analytical grade.

### 3.2. Synthesis

Sol–gel saponite, with the theoretical formula (Na)_1_._2_Si_6_._8_Al_1_._2_Mg_6_O_20_(OH)_4_·nH_2_O, was synthesized by following the procedure described by Vogels [35]. Solution A was obtained by diluting 4 g of Na_2_SiO_3_ solution in 10 mL of distilled water. A total of 1.19 g of Al(NO_3_)_3_⋅9H_2_O was dissolved in 8 mL of NaOH 2 M solution to obtain solution B. Solution B was added dropwise to solution A while stirring, and the mixture was stirred until gelation occurred. The gel stood for 1 h before dispersion in 100 mL of distilled water and heating at 90 °C. A total of 4.06 g of Mg(NO_3_)_2_⋅6H_2_O and 3.6 g of urea were dissolved in 50 mL of distilled water and added to the dispersion. The product was washed with distilled water after 20 h of reaction, then centrifuged (6000 rpm, 5 min), and dried overnight in an oven at 130 °C.

For the hydrothermal saponites, we synthesized two samples with the theoretical formulas (Na)_1_._2_Si_6_._8_Al_1_._2_Mg_6_O_20_(OH)_4_·nH_2_O and (Na)_0_._6_Si_7_._4_Al_0_._6_Mg_6_O_20_(OH)_4_·nH_2_O. Hereafter, we report the synthesis procedure adopted for the first sample. For the second one, the content of the silicon and aluminum reagents was properly scaled. Solution A was prepared by adding 5.6 mL of a Na_2_SiO_3_ solution to a pH = 13 buffer and stirring for 10 min. The buffer was obtained by dissolving 3.63 g of NaOH and 6.56 g of NaHCO_3_ in 50 mL of water. Then, a slurry was obtained by adding solution B, prepared by dissolving 2.31 g of Al(NO_3_)_3_·9H_2_O and 7.91 g of Mg(NO_3_)_3_·6H_2_O in 5 mL of water, to solution A. The slurry was stirred for 30 min, sonicated for 30 min, and then transferred to a sealed autoclave and aged for 72 h at 180 °C. The products were washed twice with distilled water and then dried overnight at 90 °C [43].

Bumetanide/clay composites were synthesized following a solution method. A total of 40 mg of BUM (80 mg for BUM/SAP-H06-H sample, see Table 2) was dissolved in 30 mL of a 1:1 H_2_O:EtOH solution and acidified with HCl to protonate the anilinic group. Then, 160 mg of clay (120 mg for BUM/SAP-H06-H sample) was added, and the dispersion was stirred for 24 h. The products were washed twice with distilled water and once with EtOH and then dried overnight in an oven at 40 °C.

A list of the investigated samples and the alphanumeric codes used is reported in Table 2.

### 3.3. Methods

#### 3.3.1. PXRD

The PXRD data were collected with a Bruker D5005 diffractometer (Bruker, Karlsruhe, Germany), CuKα radiation, a curved graphite monochromator on the diffracted beam and a scintillation detector. Each sample was mildly milled in an agate mortar to obtain fine powders. All the diffraction patterns were collected on a silicon low-background sample holder (20 mm diameter × 0.5 mm sample cavity) with a step size of 0.03° (2θ). The angular range and counting time/step depended on the sample: 2–70° (2θ) and 22 s/step for saponites, 2–35° (2θ) and 10 s/step for bumetanide, and 2–40° (2θ) and 32 s/step for saponite/bumetanide samples.

#### 3.3.2. Computational Modeling

The molecular geometry of the protonated bumetanide was optimized using the semi-empirical PM3MM method. Solvent effects were taken into account by modeling the compound in water using the integral equation formalism of the polarizable continuum model (IEFPCM). Using the same computational approach, a relaxed potential energy surface (PES) scan was performed by constraining a selected dihedral angle at 5° intervals while fully optimizing all remaining geometrical parameters at each scan point. All the calculations were carried out using the Gaussian 16 program package, revision C.01 [44], installed on Galileo100 at the CINECA facility (Italy) and at the EOS facility at the University of Pavia (Italy).

#### 3.3.3. Morphological Characterization

SEM images were obtained by a Zeiss EVO MA10 microscope (Carl Zeiss, Oberkochen, Germany) coupled with an EDS detector (X-max 50 mm^2^, Oxford Instruments, Oxford, UK) on gold-sputtered samples in an argon atmosphere. A 20 kV voltage was used. The image magnification ranged between 1.5 and 30 kX.

#### 3.3.4. Bumetanide Content

The drug loading of the different hybrids was determined by placing exactly weighed quantities of the hybrid compounds in flasks containing a known volume of a fluid in which the drug was very soluble (deionized water for BUM) and leaving them under continuous magnetic stirring. After time intervals, the UV–Vis absorbance (Lambda 25, Perkin-Elmer, Monza, Italy) was measured on a filtered portion of the fluid, and this was repeated until the readings were constant over time. A calibration curve was determined at 330 nm, in the range 0.1–4.0 mg/L, with a correlation coefficient of 0.9999; the LOQ was 5.6 μg/L. The UV spectrum of the inorganic carrier had been previously acquired to exclude possible interference with the determination of the drug.

The thermogravimetric data were collected with a Discovery TGA55 apparatus interfaced with a TRIOS data station (TA Instruments, NewCastle, DE, USA). The samples (about 5 mg) were loaded into an open aluminum pan, and the thermal treatment was carried out under N_2_ flow (3 L⋅h^−1^) at a heating rate of 10 °C⋅min^−1^. All TGA data are averages of at least three experiments.

Differential Scanning Calorimetry data were obtained by a DSC Q2000 apparatus interfaced with a TA 5000 data station (TA Instruments, Newcastle, DE, USA). The instrument was calibrated using ultrapure (99.999%) indium (m.p. = 156.6 °C; ΔH = 28.54 J g^−1^) as a standard. The samples (about 5 mg) were loaded into open standard aluminum pans, and the thermal treatment was carried out under N_2_ flow (45 mL min^−1^) at a heating rate of 10 °C min^−1^. All DSC data are averages of at least three experiments.

#### 3.3.5. ATR-FTIR

A Nicolet FT-IR iS10 Spectrometer (Nicolet, Madison, WI, USA) equipped with an ATR (attenuated total reflectance) sampling accessory (Smart iTR with ZnSe plate, Thermo Fisher Scientific, Waltham, MA, USA) was used for collecting the FT-IR spectra. The spectra were collected at different spots of each sample to assess measurement reproducibility and were obtained by co-adding 32 scans at a 4 cm^−1^ resolution in the 4000–550 cm^−1^ range.

#### 3.3.6. Solubility

The solubility was determined in triplicate using the shake flask method in distilled water at 21 °C. An excess of the different samples was poured into volumetric flasks, which were left under magnetic stirring at 200 rpm. At different times, the suspension was filtered (0.22 μm, Millipore) and properly diluted. The concentration was measured using the spectrophotometric method previously described, and the readings were repeated until equilibrium was reached.

#### 3.3.7. Wettability

The sample’s wettability was determined by contact angle θ (deg) measurements (Meter DMe-211Plus NTG Nuova Tecnogalenica, Cernusco, Italy). A 9 µL drop of fluid (deionized water, pH 1.2 simulated gastric fluid, or pH 4.5 buffer) was extruded from the needle and dropped onto the solid surface of the powder. At progressive times, photos of the drop were recorded, and its contact angle with the powder surface was measured by the software provided. Three replicas for each sample were performed.

#### 3.3.8. Dissolution Tests

The dissolution tests were performed on the drug alone and on the BUM/SAP samples previously sieved through a 230 mesh grid (63 μm) (Endecotts, London, UK) using a USP apparatus 2, with the paddle set at 50 rpm (Erweka DT-D6, Erweka GmbH, Dusseldorf, Germany), in 900 mL of deionized water according to the official monograph reported in the US Pharmacopoeia [45] at 37.0 ± 0.5 C° (three replicates). The test was performed in the same volume as the other two biorelevant fluids to simulate the in vivo condition of oral administration: simulated gastric fluid pH = 1.2 without enzymes (simulating the stomach fluid in fasted conditions) and pH 4.5 phosphate buffer to simulate gastric fed conditions (USP reagents section) [46]. All the samples contained a 2 mg dose of BUM. The drug concentrations were measured spectrophotometrically on filtered portions of the dissolution fluid. The data were processed using suitable software (Winlab V6 software, Perkin-Elmer, Monza, Italy), and the percentage of the dose was plotted as a function of time.

The tablet formulation was prepared by mixing 2 mg of BUM or a quantity of the different BUM/SAP samples corresponding to a dose of 2 mg of drug with 40 mg of microcrystalline cellulose, 40 mg of cross-linked polyvinyl pyrrolidone, 31 mg of sodium hydrogen carbonate, 15 mg of polyoxyethylene polyoxypropylene triblock copolymer, 5 mg of sodium lauryl sulfate, 1 mg of colloidal silicon dioxide and 1 mg of magnesium stearate. The different sieved components were mixed in a Turbula apparatus (Bachofen, Basel, Switzerland) for 15 min at 20 rpm. The tablets were produced using a Korsh EK0 reciprocating tablet press (Berlin, Germany) equipped with 7 mm diameter flat punches. Pharmaceutical tablets with a diameter of 7 mm require a hardness (crushing strength) between 70 N and 150 N to withstand handling, packaging, and transportation. The tabletting machine was set to achieve a value within this range; tablet thickness was checked to verify batch uniformity. The tablet hardness was 120 +/− 42 N, and the thickness was 2.8 +/− 0.2 mm.

## 4. Conclusions

New bumetanide/saponite composites were prepared to enhance the drug dissolution rate. Unlike other drug/clay systems reported in the literature, bumetanide was loaded onto synthetic saponites specifically prepared with different degrees of stacking and aluminum contents to investigate in depth the role of the clay structure on the composite properties and drug dissolution behavior. The characterization techniques highlight the co-existence of crystalline and non-crystalline bumetanide in the hybrids. Interestingly, the relative abundance of the two components can be tuned by varying the saponite stacking and drug loading. In fact, the non-crystalline form prevails in stacked saponites (hydrothermal approach), and bumetanide molecules are intercalated within the saponite interlayer space, which provides a suitable environment for drug accommodation, as supported by computational modeling. On the contrary, saponite synthesized by the sol–gel route displays a poorly stacked structure, and bumetanide preferentially segregates in crystalline form. A theoretical loading of 20% *w*/*w* results in an effective loading in the 11.50–17.80% *w*/*w* range, depending on the sample, which is quite good from a pharmaceutical viewpoint. Notably, the amount of the intercalated drug cannot be increased by raising the theoretical bumetanide content (40% *w*/*w*).

The loading mechanism is promoted by the attractive electrostatic interaction between the protonated anilinic group and the negatively charged aluminosilicate layers. Loading BUM onto saponite always increases the drug release rate from the system, likely due to the intercalation capacity of the support and the reduction in the crystalline fraction of the active ingredient. However, this technique was not sufficient to ensure complete release of the dose in sufficiently short times in all the fluids considered. This limitation was overcome by formulating the hybrid systems with suitable excipients, whereas the formulation containing the pure drug alone failed to achieve complete dose release. The combination of hybrid bumetanide/saponite samples and tablet formulation led to a successful outcome.

Overall, this study provides an appealing approach to improve the water solubility and dissolution rate of bumetanide. The chosen synthetic methods for preparing saponites are well-established and scalable routes that are suitable to obtain samples with high purity and homogeneous morphology. Drug loading, performed via a solution method, is feasible and scalable, too. Interestingly, drug solubility and dissolution behavior can be tuned by modifying clay stacking. Finally, this study demonstrates that applying a single technology to improve drug dissolution characteristics may provide remarkable improvement, but it may not be sufficient to completely resolve the problem when used by itself. However, a multidisciplinary approach, such as loading the drug onto saponite and a suitable formulation, has been shown to significantly improve the dissolution rate of bumetanide.

## Figures and Tables

**Table 2:** List and codes of the investigated samples and the amount of bumetanide used for the synthesis of the hybrid systems.

SAMPLE	BUM Amount from Synthesis (% *w*/*w*)	CODE
Saponite (hydrothermal x = 0.6)	-	SAP-H06
Saponite (hydrothermal x = 1.2)	-	SAP-H12
Saponite (sol–gel x = 1.2)	-	SAP-SG
Bumetanide	-	BUM
Bumetanide/saponite (hydrothermal x = 0.6)	20	BUM/SAP-H06
Bumetanide/saponite (hydrothermal x = 0.6)	40	BUM/SAP-H06-H
Bumetanide/saponite (hydrothermal x = 1.2)	20	BUM/SAP-H12
Bumetanide/saponite (sol–gel x = 1.2)	20	BUM/SAP-SG

**Table 3:** Total and non-crystalline weight percentages of bumetanide in hybrid samples.

Sample	BUM % *w*/*w* (UV–Vis)	BUM % *w*/*w* (TGA)	Non-Crystalline BUM % *w*/*w* (DSC)
BUM/SAP-H12	11.50	13.15	54.09
BUM/SAP-H06	12.00	12.95	89.32
BUM/SAP-H06-H	29.10	30.26	32.22
BUM/SAP-SG	17.80	17.96	28.42

**Table 4:** FT-IR band positions and assignments of BUM and BUM/SAP composites. Bold: positions and shifts discussed in the main text.

Mode	Position (cm^−1^)				
	BUM	BUM/SAP-H12	BUM/SAP-SG	BUM/SAP-H06	BUM/SAP-H06-H
ν (OH)	3422	**3419**	3422	3421	3421
ν (NH_2_)_a_	3394	**3391**	**3397**	3393	3395
ν (NH_2_)_s_	3289	**3285**	**3291**	**3287**	3289
ν (CH)	3058	**3062**	3058	**3062**	3059
ν (CH_2_) + ν (CH_3_)	2954	**2958**	2954	**2956**	2955
	2922	**2932**	2922	**2930**	**2925**
	2861	**2863**	2861	2862	2861
ν (C=O)	1691	1692	**1693**	1692	1692
NH_2 sciss_	1585	**1587**	1586	1586	1586
NH_2 rock_	1543	**1546**	1544	**1546**	**1546**
CH_2 sciss_	1506	**1508**	1507	**1508**	1507
δ (CH)_ring_ + ν (CC)_ring_	1448	-	**1446**	-	-
ν (CC)_ring_	1434	**1436**	1435	1435	1435
δ (CH)_ring_	1341	**1343**	**1346**	**1344**	**1344**
CH_2 wag_ + ν (CC)_ring_	1302	**1300**	1302	**1300**	1301
CH_2 twist_	1283	**1285**	**1285**	**1285**	1284
γ (CH)_ring_	912	**915**	913	**916**	**915**

## Data Availability

The data presented in this study are available on request from the corresponding author.

## Supplementary Materials

The following supporting information can be downloaded at: https://www.mdpi.com/article/10.3390/molecules31173015/s1, Table S1: Position of the (001) reflection, magnesite impurity content, and crystallite size of the saponite samples; Table S2: Two-theta position, intensity, *d*-spacing, and *h k l* indices and of the main peaks of the bumetanide sample; Figure S1: Crystal structure of triclinic bumetanide viewed along the *a*-axis; Figure S2. Optimized geometry (corresponding to the global minimum on the potential energy surface) of protonated bumetanide. (a) Front view and (b) side view, with the dihedral angle selected for the relaxed potential energy surface scan highlighted. Figure S3. Side views of protonated bumetanide: (a) optimized structure corresponding to the global minimum on the potential energy surface and (b) optimized structure representing a potentially accessible conformer characterized by moderately higher potential energy, resulting from the PES scan. Figure S4: TG curves of saponite samples; Table S3: Mass losses of pristine clays, bumetanide, and bumetanide/clays hybrids in the 200–450 °C temperature range; Figure S5: DSC curves of saponite samples; Table S4: Melting process of bumetanide: T_onset_, ΔH, and crystalline bumetanide weight percentage; Figure S6: FT-IR spectra of saponite samples; inset: magnification of the antisymmetric Si-O-Si stretching mode; Table S5: FT-IR band positions and assignments of bumetanide; Figure S7: Contact angle test: images of the droplets of the three fluids deposited on the surface of the BUM and of different BUM/SAP samples at a time of 30 s.
