# Supplementary material for: Bumetanide/Synthetic Saponite Composites: Performant Systems for Enhanced Drug Dissolution Rate
Source: Molecules. 2026 Aug 28;31(17):3015. doi: 10.3390/molecules31173015 (PMC13567011; doi:10.3390/molecules31173015)
Supplement: Supplementary file 1 [file molecules-31-03015-s001.zip › molecules-4433303-supplementary.pdf]

Bumetanide/synthetic saponite composites: performant systems for enhanced drug dissolution rate

Valeria Friuli [1], Doretta Capsoni [2], Lauretta Maggi [1], Giovanna Bruni [2], Elena Cassera [3], and Claudia Urru [2,*]

[1] Department of Drug Sciences, University of Pavia, Via Taramelli 12, 27100 Pavia, Italy; valeria.friuli@unipv.it (V.F.); lauretta.maggi@unipv.it (L.M.)
[2] Department of Chemistry, Physical Chemistry Section & C.S.G.I. (Consorzio Interuniversitario per lo Sviluppo dei Sistemi a Grande Interfase), University of Pavia, Via Taramelli 12, 27100 Pavia, Italy; doretta.capsoni@unipv.it (D.C.); giovanna.bruni@unipv.it (G.B.)
[3] Department of Chemistry, University of Pavia, Via Taramelli 12, 27100 Pavia, Italy; elena.cassera01@universitadipavia.it (E.C.)
* Correspondence: claudia.urru01@universitadipavia.it (C.U.)

Table S1. Position of the (001) reflection, magnesite impurity content, and crystallite size of the saponite samples.

Sample	(001) 2theta position (°)	Magnesite content (%w/w)	Crystallite size (nm)
SAP-H12	7.07	4.0	11.1
SAP-H06	7.09	1.0	10.2
SAP-SG	-	-	5.0

Table S2. Two-theta position, intensity, *d*-spacing, and *h k l* index of the main peaks of the bumetanide sample.

**2θ (°)**	4.58	9.18	9.69	11.37	12.27	13.80	14.37	17.43	18.09	18.45	19.51	19.66	20.20
**I (%)**	100.00	10.83	1.50	0.47	1.33	2.11	1.32	0.38	3.11	4.10	2.12	1.00	0.48
***d* (Å)**	19.28	9.63	9.12	7.78	7.21	6.41	6.16	5.08	4.90	4.80	4.55	4.51	4.39
h k l	0 0 1	0 0 2	0 1 0	0 1 -1	0 1 2	0 0 3	0 1 -2	1 0 1	0 1 -3	0 0 4	0 1 4	1 1 1	0 2 2

**2θ (°)**	20.71	21.07	21.25	22.12	22.90	23.68	24.19	24.73	27.61	28.24	29.02	31.01	31.37
**I (%)**	2.58	0.76	0.44	1.86	0.63	0.95	1.81	0.53	1.43	0.63	0.47	0.65	0.78
***d* (Å)**	4.29	4.21	4.18	4.02	3.88	3.75	3.68	3.60	3.23	3.16	3.07	2.88	2.85
h k l	0 2 -1	1 -1 1	1 0 3	0 2 3	0 2 -2	0 1 5	1 0 4	1 1 4	1 0 5	1 2 -2	0 3 1	0 1 -6	1 1 6

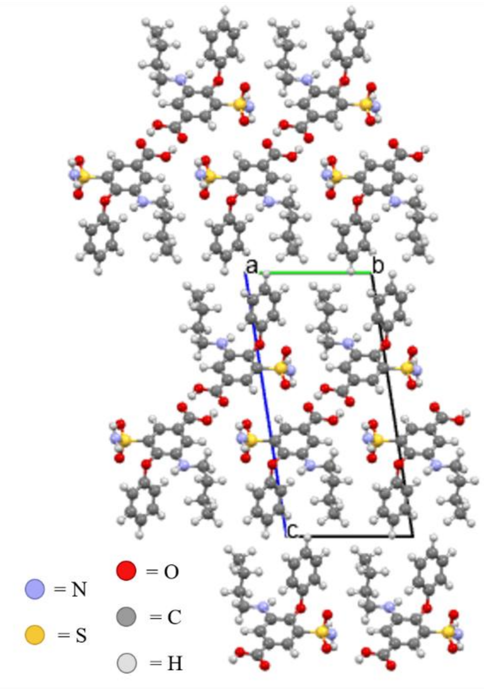

Figure S1. Crystal structure of triclinic bumetanide viewed along the a-axis.

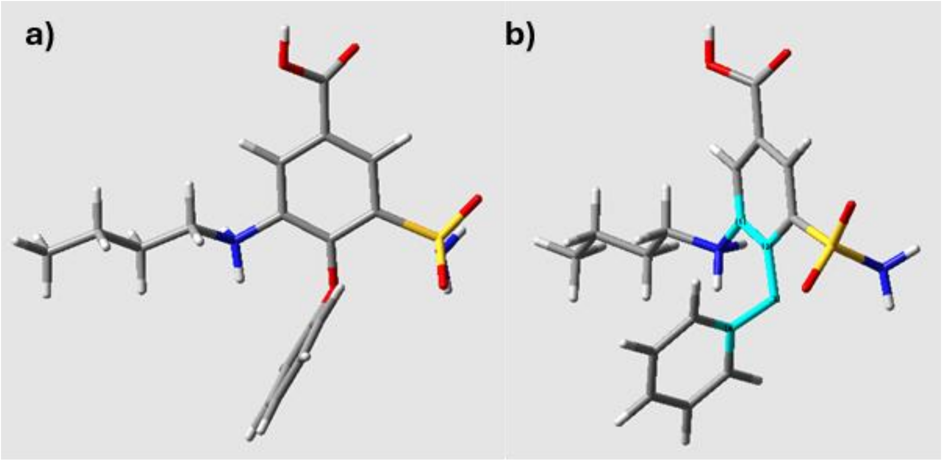

Figure S2. Optimized geometry (corresponding to the global minimum on the potential energy surface) of protonated bumetanide. a) front view and b) side view, with the dihedral angle selected for the relaxed potential energy surface scan highlighted.

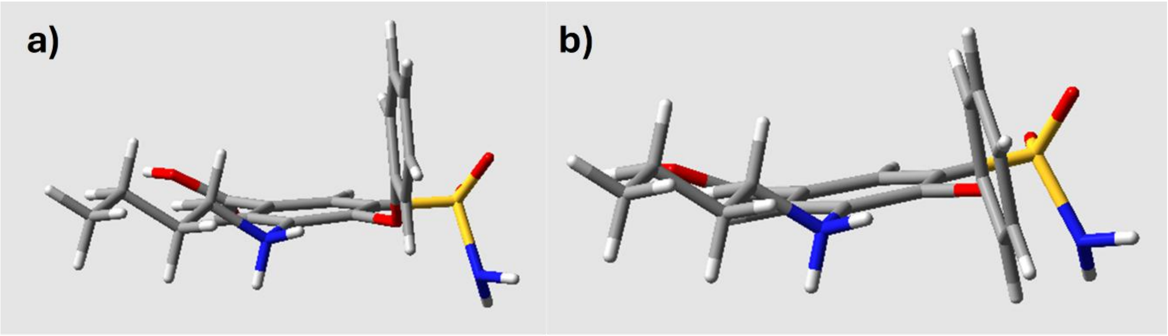

Figure S3. Side views of protonated bumetanide: a) optimized structure corresponding to the global minimum on the potential energy surface, and b) optimized structure representing a potentially accessible conformer characterized by moderately higher potential energy, resulting from the PES scan.

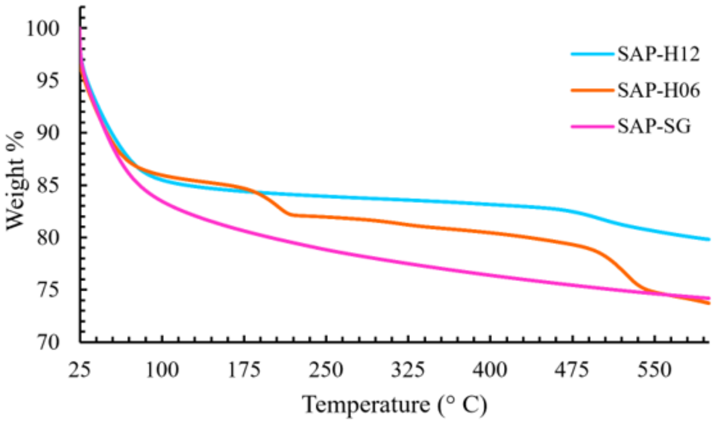

Figure S4. TG curves of saponite samples.

Table S3. Mass losses of pristine clays, bumetanide, and bumetanide/clays hybrids in the 200-450°C temperature range and bumetanide weight percentage of the composite samples.

Sample	Δm 200 – 450 °C (%w/w)	Drug contribution (%w/w)*	Effective loading (% w/w)**
BUM	80.954	-	-
SAP-H12	1.044	-	-
SAP-H06	1.608	-	-
SAP-SG	3.625	-	-
BUM/SAP-H12	11.690	10.646	13.15
BUM/SAP-H06	12.081	10.473	12.95
BUM/SAP-H06-H	26.099	24.491	30.26
BUM/SAP-SG	18.161	14.536	17.96

$$* \, Drug\ contribution = \Delta m_{hybrid} - \Delta m_{clay}$$

$$** \, Effective\ loading = \frac{Drug\ contribution}{\Delta m_{drug}} \cdot 100$$

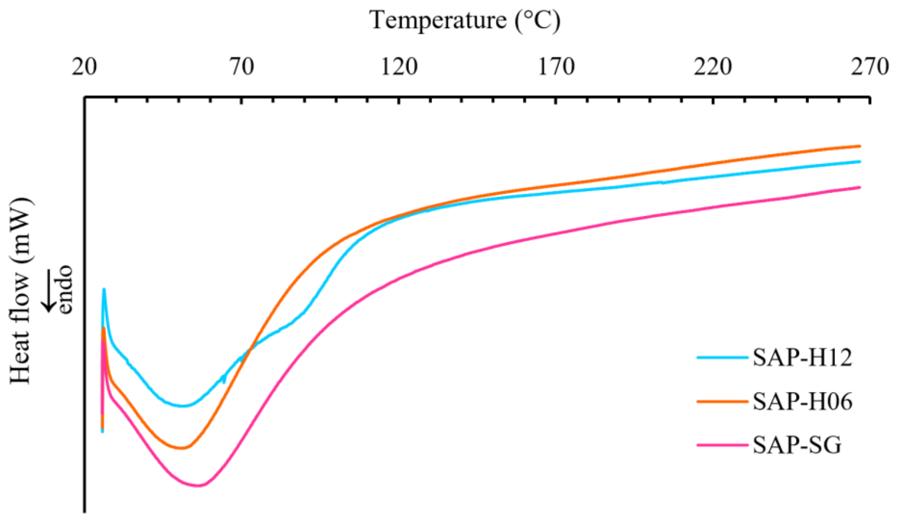

Figure S5. DSC curves of saponite samples.

Table S4. Melting process of bumetanide: T_{onse} , ΔH, and crystalline bumetanide weight percentage.

	T_{onset} (°C)	ΔH_m (J/g)	Crystalline Bumetanide (%w/w)
BUM	234.65	123.30	100.00
BUM/SAP-H12	232.35	6.51	5.28
BUM/SAP-H06	231.76	1.58	1.28
BUM/SAP-H06-H	232.57	24.32	19.72
BUM/SAP-SG	234.02	15.71	12.74

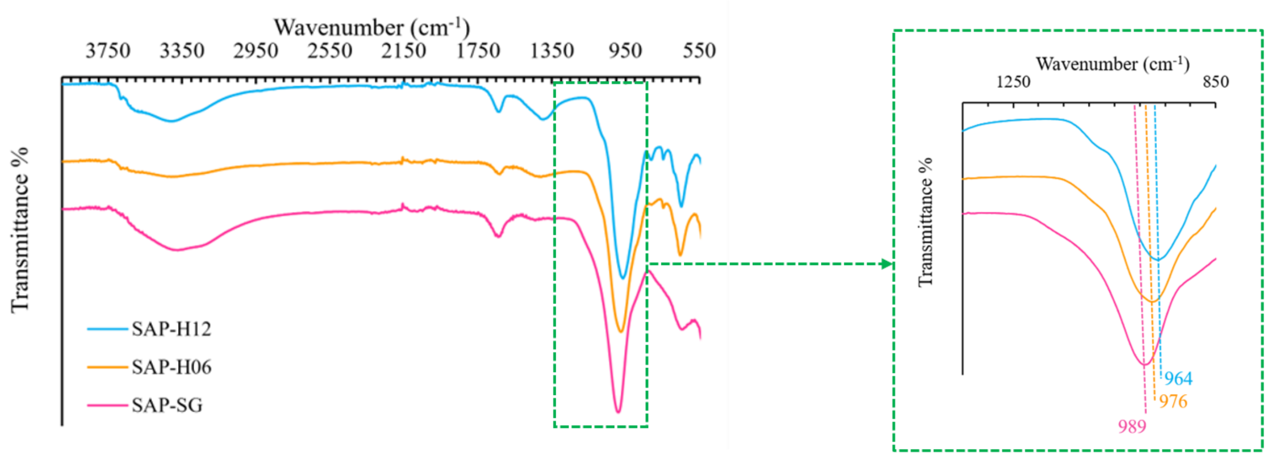

Figure S6. FT-IR spectra of saponite samples; inset: magnification of the antisymmetric Si-O-Si stretching mode.

Table S5. FT-IR band positions and assignments of bumetanide.

Assignment	Position (cm^{-1})	Assignment	Position (cm^{-1})	Assignment	Position (cm^{-1})
ν (OH)	3422	NH_2 sciss	1585	ν $(CC)_{ring}$ + ν (CO)	1104
ν $(NH_2)_a$	3394	CH_2 sciss	1506	ν (CN)	1040
ν $(NH_2)_s$	3289	δ $(CH)_{ring}$ + ν $(CC)_{ring}$	1489	δ ring + ν $(CC)_{ring}$	1002
ν (CH)	3058		1448	CH_2 twist + CH_2 rock	955
ν (CH_2) + ν (CH_3)	2965		1200	γ $(CH)_{ring}$	912
	2954	CH_2 sciss + δ (CCH_3)	1468		892
	2922	CH_2 wag + δ (CCH_3)	1364		741
	2872	δ $(CH)_{ring}$	1341	CH_2 rock + δ (CCH_3)	882
	2861	ν $(S=O)_a$ + ν $(CC)_{ring}$	1331	NH_2 wag + ν (NS)	854
	2645	CH_2 wag + ν $(CC)_{ring}$	1302	CO wag + γ $(CH)_{ring}$	794
ν (C=O)	1691	CH_2 twist	1283	CO wag	772
ν $(CC)_{ring}$	1603	CH_2 wag	1251	CH_2 rock	733
	1434	ν $(CO)_{connect}$	1217	δ ring + τ ring	717
NH_2 rock	1543	ν (CO) + δ COH	1155		628
	1082	ν $(S=O)_s$	1145	NH_2 wag + SO_2 wag	685

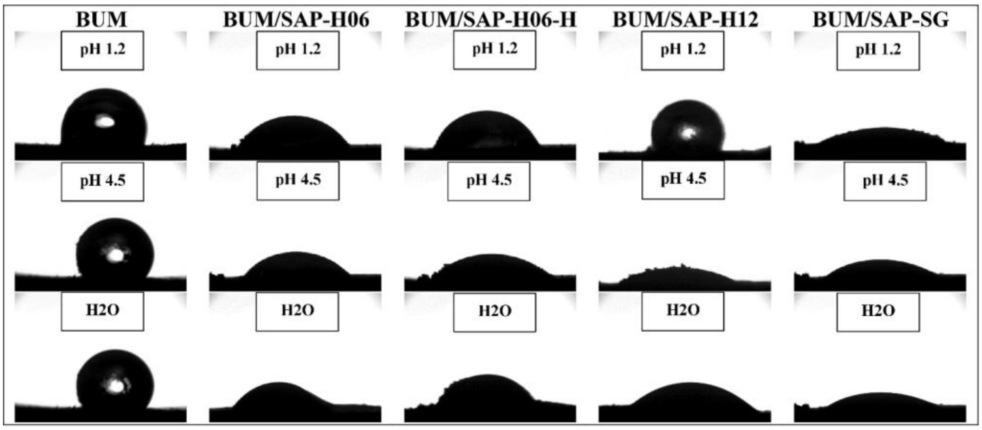

Figure S7. Contact angle test: images of the droplets of the three fluids deposited on the surface of the BUM and of different BUM/SAP samples, at the time of 30 seconds.

The contact angle was determined using the same fluids mentioned for the dissolution test: deionized water, simulated gastric fluid (to simulate a fasted gastric environment), and phosphate buffer pH 4.5 (to simulate a fed gastric environment). The fluids were prepared according to the "Reagents. Solutions/Buffer solutions" section of the USP [1].

References

1. Reagents. Solution/Buffer Solutions. In *United States Pharmacopeia (USP43-NF38)*; United States Pharmacopeial Convention, Inc.: Rockville, MD, USA, 2023; p. 6223.
